# Diversification of *Sinorhizobium* populations associated with *Medicago polymorpha* and *Medicago lupulina* in purple soil of China

**DOI:** 10.3389/fmicb.2022.1055694

**Published:** 2023-01-04

**Authors:** Mingxing Tang, Haoyu Wang, Xin Qi, Teng He, Bin Zhang, Entao Wang, Miao Yu, Beinan Wang, Fang Wang, Zhongkuan Liu, Xiaoyun Liu

**Affiliations:** ^1^Key Laboratory of Microbial Diversity Research and Application of Hebei Province, College of Life Science, Engineering Laboratory of Microbial Breeding and Preservation of Hebei Province, Institute of Life Science and Green Development, Hebei University, Baoding City, China; ^2^Departamento de Microbiología, Escuela Nacional de Ciencias Biológicas, Instituto Politecnico Nacional, Mexico City, Mexico; ^3^Key Laboratory of State Forestry Administration for Biodiversity Conservation in Southwest China, Southwest Forestry University, Kunming City, China; ^4^Institute of Agricultural Resources and Environment, Hebei Academy of Agriculture and Forestry Sciences, Shijiazhuang, China

**Keywords:** *Medicago*, rhizobia, diversity, phylogeny, plant host, soil nutrient

## Abstract

The double selection of environment adaptation and host specificity forced the diversification of rhizobia in nature. In the tropical region of China, *Medicago polymorpha* and *Medicago lupulina* are widely distributed, particularly in purple soil. However, the local distribution and diversity of rhizobia associated with these legumes has not been systematically investigated. To this end, root nodules of *M. polymorpha* and *M. lupulina* grown in purple soil at seven locations in Yunnan Province of China were collected for rhizobial isolation. The obtained rhizobia were characterized by RFLP of 16S–23S rRNA intergenic spacer, BOXAIR fingerprinting, and phylogeny of housekeeping and symbiosis genes. As result, a total of 91 rhizobial strains were classified into species *Sinorhizobium medicae* and *S. meliloti*, while three *nodC* gene types were identified among them. *S. medicae* containing *nodC* of type I was dominant in farmlands associated with *M. polymorpha*; while *S. meliloti* harboring *nodC* of type III was dominant in wild land nodulated by *M. lupulina*. For both rhizobial species, greater genetic diversity was detected in the populations isolated from their preferred host plant. A high level of genetic differentiation was observed between the two *Sinorhizobium* species, and gene flow was evident within the populations of the same species derived from different soil types, indicating that rhizobial evolution is likely associated with the soil features. To examine the effects of environmental features on rhizobial distribution, soil physicochemical traits and rhizobial genotypes were applied for constrained analysis of principle coordinates, which demonstrated that soil features like pH, nitrogen and sodium were the principle factors governing the rhizobial geographical distribution. Altogether, both *S. medicae* and *S. meliloti* strains could naturally nodulate with *M. polymorpha* and *M. lupulina*, but the rhizobium-legume symbiosis compatibility determined by both the host species and soil factors was also highlighted.

## Introduction

Root and/or stem nodules on leguminous plants induced by the rhizobia formed the symbiotic nitrogen-fixing system, which is the most significant and efficient biological nitrogen fixation (BNF) system and contributes to one quarter of the global nitrogen fixation (Biswas and Gresshoff, 2014). This system could satisfy a major part or all of the nitrogen (N) requirement of the host legumes, as well as supply the N nutrient to the adjacent/subsequent plants (Wang et al., 2019). The rhizobium-legume symbiosis is specific and it is regulated by the molecular recognition between the partners: with flavonoids in root exudates and Nod factor synthesized by rhizobia as the primary molecular signals (Wang et al., 2018). For soybean rhizobia, the host specificity is mostly determined by the nodulation (*nod*) genes localizing in the mobile genetic elements, either as symbiotic plasmids in fast growing rhizobia (*Sinorhizobium*) or as symbiotic islands in chromosomes of slow-growing rhizobia (*Bradyrhizobium*; Wang et al., 2018). Therefore, distinct rhizobial species harboring similar *nod* genes could nodulate the same host legume. Moreover, the biogeographic patterns or structure of rhizobial communities can be shaped by the double selection of the host (for *nod* gene background) and the soil conditions (for adaptation genes in chromosome; Wang et al., 2019). Accordingly, the introduced legumes might contract rhizobia differing from that in their native habitats. For example, chickpea (*Cicer arietinum* L.) mainly nodulated with *Mesorhizobium ciceri* and *Mesorhizobium mediterraneum* at its origin center, but with *Mesorhizobium muleiense* and *Mesorhizobium wenxiniae*, and several unidentified *Mesorhizobium* species in China (Zhang et al., 2012, 2014, 2020). In this case, the nodulation genes specific to chickpea might have been transferred from *M. ciceri* to the other soil bacteria after this plant was introduced. Therefore, study on the rhizobia associated with the exotic legumes might offer us information about the rhizobial evolution following the migration of their hosts.

Similar to chickpea, the annual legume species *Medicago polymorpha* (commonly named Burr medic) was introduced as a winter green manure cultured in rice field of semi-arid and humid regions in China. This legume can grow in soils with pH ranging from 5.0 to 8.5 and it was nodulated only by *Sinorhizobium medicae* in the Mediterranean countries (France and Italia) (Brunel et al., 1996; Garau et al., 2005) and in Mexico (Silva et al., 2007). In pasture land of Australia, *M. polymorpha* forms root nodules with both *Sinorhizobium meliloti* and *S. medicae* with sub-optimal efficiency in nitrogen fixation (Charman and Ballard, 2004). The ineffective or sub-effective symbiosis between.

*S. meliloti* and *M. polymorpha* was also reported in other studies (Garau et al., 2005; Villegas et al., 2006; Alías-Villegas et al., 2015). Based on the previous studies, *M. polymorpha* seems a compatible host for *S. medicae* and *S. meliloti* in newly colonized regions (Charman and Ballard, 2004) and in laboratory (Villegas et al., 2006; Silva et al., 2007; Alías-Villegas et al., 2015). So, a study on rhizobia associated with *M. polymorpha* in relation to the soil physicochemical features might give some useful information about the effects of interactions among the rhziobia, host plants and environmental factors on the biogeography and evolution of these rhizobia.

It has been described that edaphic properties significantly influence the interaction of plant and rhizobia, which in some way, constrain the successful development of nodulation in a new habitat (Denton et al., 2007). *S. medicae* strains are mostly acid-tolerant and associate with annual medics such as *M. polymorpha*, *M. arabica* and *M. murex*; whereas *S. meliloti* was predominantly associated with *M. littoralis* and *M. tornata* grown in soils with alkaline or neutral pH (Garau et al., 2005; Reeve et al., 2010). Although *Medicago sativa* forms nodules with *S. meliloti* in natural conditions, its growth in acidic soils was also significantly improved by inoculating *S. medicae* under laboratory conditions (Ramírez-Bahena et al., 2015). All these results imply that the dominant association of *S. medicae* with *M. polymorpha* might be related to the natural distribution of this legume plant in acid soil, despite that alkaline-tolerant (pH = 8.0) *S. medicae* strain has also been reported (Ardley et al., 2015).

In the present work, we analyzed the diversity and biogeography of rhizobia associated with *M. polymorpha* in field with purple soil in Yunnan Province of China. Since *Medicago lupulina*, an annual medic normally nodulated with *S. meliloti* and *S. fredii* (Kan et al., 2007; Li et al., 2012), naturally grows in the same region with *M. polymorpha,* it was also included here to compare its rhizobial profile with that of *M. polymorpha.* The aims of this study were: (1) to investigate the diversity and geographical distribution of rhizobia associated with these two *Medicago* species; (2) to explore the interaction between rhizobia and legumes mediated by soil traits.

## Materials and methods

### Nodule collection and soil physicochemical characterization

Root nodules of *M. polymorpha* and *M. lupulina*, as well as soil samples in the root zone of these plants, were collected from farmlands and wild fields on 7 locations (Dabanqiao, Jinshan, Yanzhan, Cangling, Jiangchuan, Yinjiang and Yiliang towns) with 39 sample sites in Yunnan (Supplementary Figure S1). In each sampling site, 5–10 plants were sampled for collecting the effective (red colored) root nodules. Soils were collected compositely from the sampled sites (5–20 cm in depth) and were mixed as a single sample for each site. Complete nodules were dissected immediately from rinsed roots and stored over dehydrated silica gel in sealed drying tube until their use for rhizobial isolation in the laboratory. Soil samples were ground and passed through 2-mm mesh screens for determining the physicochemical properties. Soil available nitrogen (AN), available phosphate (AP; using Bray’s hydrochloric acid fluoride ammonium by extraction method), and available potassium (AK; by ammonium acetate extraction plus flame photometry) were determined with the standard procedures (Du and Gao, 2006). Soil pH was measured using a pH meter (Mettler Toledo) by suspending 5 g of soil in 5 ml of distilled water, and organic matter (OM) was measured using the potassium dichromate volumetric method (Du and Gao, 2006). Soil contents of Cl^−^ and HCO_3_^−^ were determined by silver nitrate titration and potentiometer titration, respectively. Concentrations of Ca^2+^, Mg^2+^, Na^+^, and SO_4_^2−^ were measured using inductively coupled plasma atomic emission spectrometry.

### Rhizobial isolation and its/BOXAIR fingerprinting

After rehydrated in sterile water for 1–2 h, the stored nodules were surface sterilized by immerging in 95% (v/v) ethanol for 30 s and in 0.2% mercuric chloride for 2 to 3 min (depending on the nodule diameter), following by rinsing six times in sterile water. Then, the nodules were crushed individually and the extract from each nodule was streaked on plates of yeast-mannitol agar (YMA, pH = 6.8–7.2) and incubated at 28°C for 2–5 days (Vincent, 1970). The obtained bacterial colonies were purified by repeatedly streaking on the same medium plates. Cultures of pure isolates were stored in YM broth supplied with 30% (w/v) of glycerol at −70°C.

For each isolate, as well as the type strains of *S. meliloti* USDA 1002^T^ and *S. medicae* A321^T^, genomic DNA was extracted by guanidine isothiocyanate method using the FastPure Bacteria DNA isolation Mini kit (Nanjing Vazyme Biotech CO., Ltd) from 5 ml of overnight culture in YM broth (Vincent, 1970). Using the DNA extract as template and the primer BOXAIR (5´-CTA CGG CAA GGC GAC GCT GACG-3′; Versalovic et al., 1994), BOXAIR-PCR was performed in a total volume of 25 μl reaction mixture with the procedure of Nick et al. (1999). The PCR products were separated by electrophoresis in 1.5% (w/v) agarose gels containing ethidium bromide (0.5 μg mL^−1^) and were photographed under UV light. The BOXAIR profiles were distinguished by their different band patterns, e.g., the isolates sharing the same BOXAIR pattern were treated as clones of the same strain. For analysis of restriction fragment length polymorphism (FRLP), 16S–23S rRNA intergenic spacer (IGS) was amplified in 25 μl volume with primers FGPS1490 (5′-TGC GGC TGG ATC ACC TCC TT-3′) and FGPS132 (5′-CCG GGT TTC CCC ATT CGG-3′) by the corresponding PCR protocol (Laguerre et al., 1996). Aliquot of 5 μl of the PCR products were used to verified the IGS amplification (about 900 bp) by electrophoresis in 1% (w/v) agarose gel. Aliquot of 5–10 μl, depending on the concentration, was digested separately with the restriction endonucleases *Hae*III (GG|CC), *Rsa* I (GT|AC), *Hif* I (G|ANTC) and *Msp* I (C|CGG; Laguerre, 1994) at 37°C for 6 h, as specified by the manufacturer with an excess of enzyme (5 U per reaction). The restriction fragments were separated by horizontal electrophoresis in agarose (2%, w/v) gels (14 cm in length) at 80 V for 3 h and were visualized by staining with ethidium bromide. Strains or isolates with different RFLP patterns were designated into distinct IGS types.

### Nodulation assays

A total of 32 representative strains were used in the nodulation tests that were selected according to their IGS types, origins of host plants, sampling sites, and BOXAIR patterns as described previously (Liu et al., 2020). This dereplication strategy was applied for checking the effects of sampling regions, origin of host species, and genomic types (backgrounds) on the compatibility of rhizobia with the host species under laboratory conditions. *M. polymorpha* (*M. lupulina* seeds were not acquired) seeds were scarified using concentrated sulfuric acid for 10 min, rinsed several times with sterile water, and then surface-sterilized in 3.2% (w/v) sodium hypochlorite followed by several rinses with sterile water. They were vernalized on 0.8% water-agar at 4°C for 3 days, and then germinated at 28°C until the seedlings developed roots of 0.5–1 cm in length. Two seedlings were transplanted into a sterile glass tubes (30 × 200 cm) half-filled with sterilized nitrogen-free plant nutrient solution (Vincent, 1970) in 0.8% agar. The seedlings were then inoculated separately with 0.1 ml YM broth culture of each test strain (about 10^8^ cells mL^−1^). Five replicates were used and blank controls without inoculation were included. The plants were placed in a growth cabinet under conditions described previously (Zhang et al., 2012). Plants were checked for nodule formation at 35 d after inoculation and BOXAIR-PCR was performed for nodule crushes to verify the nodule occupation by inoculant strains.

### Phylogenetic analyses of housekeeping genes and symbiotic genes

For determining the species affiliation of the isolates, 25 representative strains covering all the IGS-RFLP genotypes, both host species, all sampling sites and the main BOXAIR-PCR patterns were chosen for gene sequencing and phylogenetic analyses as previous described by Liu et al. (2020). The 16S rRNA gene was amplified by PCR as described previously with the primers fD1 (5′-AGA GTT TGA TCC TGG CTC AGA-3′) and rD1 (5′-AAG GAG GTG ATC CAG CC-3′; Weisburg et al., 1991). Multilocus sequence analysis (MLSA) based on the five housekeeping genes *atpD* (encoding for the ATP synthase beta-chain), *recA* (recombinase A), *glnA* (glutamine synthetase I), *dnaK* (Chaperone protein DnaK), and *gyrB* (DNA gyrase subunit B) was also performed to differentiate rhizobial species (Martens et al., 2007, 2008). The primer pairs recA41F/recA640R described by Vinuesa et al. (2005), glnA144F/glnA1142R and dnaK1466F/dnaK1777R described by Martens et al. (2007), atpD352F/atpD871R and gyrB343F/gyrB1043R described by Martens et al. (2008) were used to amplify the corresponding genes in 25 μl volume by PCR. The PCR products were checked by electrophoresis in 1% (w/v) agarose gel. After purification with the Solarbio DNA kit (Beijing Solarbio Science & Technology Co., Ltd.), the amplicons were sequenced commercially in Beijing Genomics Institute (BGI) using the same primers for PCR. The sequences acquired in this study were aligned with those from type strains of the related bacterial species obtained from the NCBI database by blasting, using Clustal W (Thompson et al., 1997). Maximum likelihood phylogenetic trees were constructed for 16S rRNA genes and for the concatenated sequences of the five housekeeping genes, and the trees were bootstrapped with 1,000 pseudo-replicates using Mega 6.1 (Tamura et al., 2013). As suggested in previous reports, similarities of 98.7% in 16S rDNA sequences and 95% in MLSA (de Lajudie et al., 1994) were taken as the thresholds for species identification.

To estimate the symbiovar of the isolates, fragments of *nifH* gene (about 800 bp) and *nodC* gene (about 700 bp) were amplified for the 25 representatives with primer pairs nifHF/nifHR and nodCF540/nodCR1160, respectively, using the protocols of Laguerre et al. (2001). The visualization, purification and sequencing of *nifH* and *nodC* amplicons were performed as that mentioned for the housekeeping genes. All of the acquired nucleotide sequences were used for alignment with related genes extracted from GenBank database by Blast, and for construction of the phylogenies using the same methods for the keeping housekeeping genes.

All the obtained nucleic acid sequences were submitted in GenBank database under the accession numbers MT863814-863838 for *nodC*, MT863855-863858, MH423019-423036 and MW250452-250454 for *nifH*, while the accession numbers for other genes are listed in Supplementary Table S1 and in the corresponding figures.

### Data analysis

The genetic differentiation for rhizobial species distributed in farmland and wild fields determined were calculated by DnaSP 5 software (Librado and Rozas, 2009) for the representative strains in MLSA. And the gene diversity and Shannon diversity index (H′; Shannon and Weaver, 1949) of total test strains were estimated by using the Popgene software 3.1 resourced from the binary dataset converted from BOXAIR profiles. The genetic differentiation levels (Kst*; Hudson et al., 1992a,b) between rhizobial communities and populations of the same species in farmlands and wild field were calculated. Correlations between soil characteristics, spatial and climates with rhizobial IGS types were analysed with constrained analysis of principle coordinates (CAP). Based on selection of the environmental factors (Anderson and Willis, 2003), only variables that significantly increase the explained variance (*p* < 0.05) were used for CAP analysis.

## Results

### Genetic diversity of rhizobial strains

Totally, 91 rhizobial isolates were obtained from 39 sampling sites in 7 locations (Supplementary Figure S1), with 70 were from *M. polymorpha* and 21 were from *M. lupulina* (Table 1). All of these strains were divided into five IGS types by IGS–RFLP analysis and displayed 39 different BOXAIR fingerprinting patterns (Table 1). The IGS type A containing 19 BOXAIR patterns was composed of 63 strains from *M. polymorpha* collected in five locations, and 4 strains from *M. lupulina* sampled in Yiliang of Kunming (Table 1). The IGS type C was represented by 24 strains from both *M. polymorpha* and *M. lupulina* with 17 BOXAIR patterns. The IGS types B, D and E were minor groups with only one or two strains included, and each strain had its own specific BOXAIR pattern.

**Table 1:** The strains isolated from *Medicago* spp. in this study and references strains and host resources.

Strains*	Numbers of isolates	Host	Types of IGS/*nodC*	BOXAIR patterns	Sampling location	Ecosystem
***Sinorhizobium medicae* genotype I (*nodC* type 2)**						
**SWF65100**, SWF65103, **SWF65105**, **SWF65107**	**4**	*M. polymorpha*	A/I	a, b, c	Dabanqiao of Kunming City	Farmland
SWF67395, SWF67451, SWF67455, **SWF67456**, **SWF67457**, **SWF67462**, SWF67463, **SWF67464**, SWF67466, SWF67470, SWF67477, SWF67479, SWF67480, SWF67481, SWF67350, SWF67356, **SWF67489**, SWF67492, **SWF67494**	19	*M. polymorpha*	A/I	d, e, f, g	Jinshan of Lufeng Town	Farmland
**SWF67394**, SWF67397, SWF67398, SWF67400, **SWF67403**, SWF67407, **SWF67409**, **SWF67410**, SWF67412, **SWF67413**, SWF67414, SWF67416, SWF67417, **SWF67418**, SWF67421, SWF67422, **SWF67423**, SWF67424, SWF67425, SWF67431, **SWF67432**, **SWF67436**, SWF67438, SWF67441, SWF67443, SWF67446, SWF67447	27	*M. polymorpha*	A/I	h, i, j, k, l, m, n, o, p	Yaozhan of Lufeng Town	Farmland
SWF67303, **SWF67448**, SWF67498, SWF67499, **SWF67500**, **SWF67501**, SWF67503, **SWF67347**	8	*M. polymorpha*	A/I	i, q, i, l, r	Cangling of Chuxiong City	Farmland
**SWF67405**	1	*M. polymorpha*	A/I	o	Yaozhan of Lufeng Town	Wild field
SWF67343, SWF67370	2	*M. lupulina*	A/I	i, r,	Yiliang of Kunming City	Wild field
**SWF65116**, **SWF66320**	2	*M. lupulina*	A/I	s,t	Yiliang of Kunming City	Farmland
***S. medicae* rDNA type II**						
**SWF67497**	1	*M. polymorpha*	B/I	u	Cangling of Chuxiong City	Wild field
***Sinorhizobium meliloti* genotype I (*nodC* type 3)**						
SWF67521, SWF67524	2	*M. polymorpha*	C/III	v,w	Yinjiang of Dehong City	Farmland
SWF67526, SWF67527, SWF67528, SWF67529, SWF67534, SWF67537	6	*M. polymorpha*	C/III	x,y,z	Yinjiang of Dehong City	Farmland
SWF66326	1	*M. lupulina*	C/III	aa	Jiangchuan of Yuxi City	Wild field
SWF66437	1	*M. lupulina*	C/III	ab	Yiliang of Kunming City	Wild field
SWF67486, **SWF67487**, SWF67344	3	*M. lupulina*	C/III	ac, ad, ae	Cangling of Chuxiong	Wild field
SWF66329, **SWF66436**	2	*M. lupulina*	C/III	af, ag	Yiliang of Kunming City	Wild field
SWF67371, SWF67373, SWF67383, SWF67393	4	*M. lupulina*	C/III	ah, ai	Jinshan of Lufeng Town	Wild field
SWF65112, **SWF65114**, **SWF65115**, HBU65332, HBU65334	5	*M. lupulina*	C/III	aj, ak	Yiliang of Kunming City	Farmland
***S. meliloti* genotype II (*nodC* type 2)**						
SWF67522, **SWF67523**	2	*M. polymorpha*	D/II	al	Yinjiang of Dehong City	Wild field
***S. meliloti* genotype III (*nodC* type 2)**						
**SWF66332**	1	*M. lupulina*	E/II	am	Yiliang of Kunming City	Wild field
*R. leguminoarum* USDA2370^T^		*Pisum sativum*				
*R. leguminosarum* 162 K68		*Trifolium pratens`*				
*S. meliloti* USDA1002^T^		*M. sativa*		-/II		
*S. medicae* USDA1037^T^		*M. truncatula*		-/I		

* The 25 strains highlighted with grey color were used in sequencing analyses; and the 32 bold marked stains were used in nodulation test.

### Symbiotic performance of representative strains on *Medicago polymorpha*

In nodulation assays (Table 1), all the plants inoculated with the 32 selected strains formed nodules on roots of *M. polymorpha* regardless of their original hosts (*M. polymorpha* or *M. lupulina*). Based on the patterns of BOXAIR-PCR of nodule crushes, most of the plants (31/32) were nodulated by the corresponding inoculants. Notably, two *S. meliloti* strains, SWF65114 and SWF65115, originally isolated from *M. lupulina* plants induced nodules on *M. polymorpha* with lower efficiency than those inoculated with *S. medicae*, with mean nodules of 2.9–4.6 compared to 5.5–6.5 (*p* < 0.01, Table 2), and the mean dry weight of 0.39–0.58 g per plant (35 d of growth) compared to 0.97–1.31 g (*p* < 0.001, Table 2).

**Table 2:** The nodulation efficient test on *M. polymorpha.*

Strains	Original hosts	Dry weight		Nodule numbers	
		Mean	STD	Mean	STD
*S. meliloti* SWF66332	*M. lupulina*	0.28	0.07	3.4	0.55
*S. meliloti* SWF67487	*M. lupulina*	0.31	0.072	2.3	0.57
*S. meliloti* SWF66436	*M. lupulina*	0.25	0.05	2	0
*S. meliloti* SWF65115	*M. lupulina*	0.39	0.03	2.9	0.23
*S. meliloti* SWF65114	*M. lupulina*	0.58	0.063	4.1	0.91
*S. meliloti* SWF67523	*M. polymorpha*	0.33	0.026	2.8	0.41
*S. medicae* SWF67405	*M. polymorpha*	0.93	0.148	4.5	0.5
*S. medicae* SWF65107	*M. polymorpha*	1.31	0.0867	5.5	0.51
*S. medicae* SWF67403	*M. polymorpha*	0.78	0.0265	4	0.83
*S. medicae* SWF67403	*M. polymorpha*	0.97	0.0458	6.5	0.51
*p*-value		<0.001***		<0.01**	

The symbols ** and *** means different significant.

### Phylogenies of housekeeping genes and species affiliation

**Figure 1:**
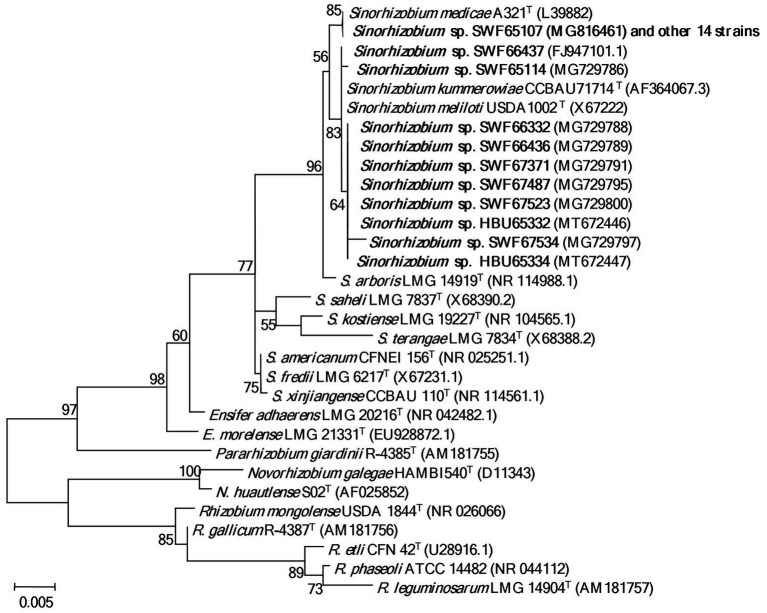
Phylogenetic tree of 16S rDNA (1,363 bp) showing the genus affiliation of rhizobia isolated from *Medicago* species in Yunnan. The tree was constructed with the Maximum likelihood method. Bootstrap values (from 1,000 replicates) > 50% are indicated in the nodes. Bar represents 0.5% substitutions per site. Strains in bold letters are obtained in this study.

**Figure 2:**
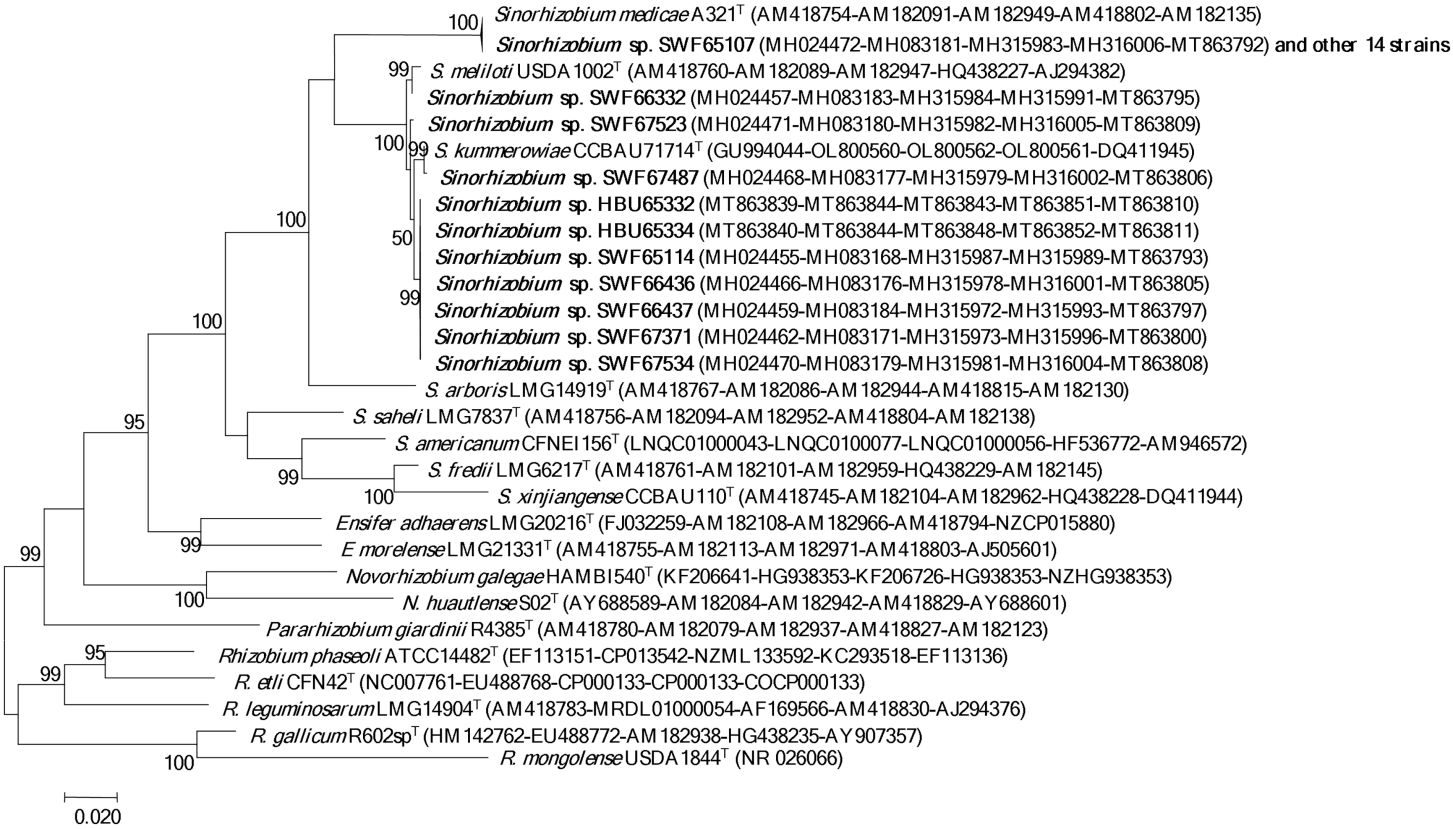
Phylogenetic tree of 5 house-keeping genes (*atpD, dnaK, glnA, gyrB* and *recA*， 2,735 bp) showing the species affiliation of the rhizobia isolated from *Medicago* species in Yunnan. The tree was constructed with the Maximum likelihood method. Bootstrap analysis was performed with 1,000 replicates and bootstrap values >5% are indicated in the nodes. Bar presents 2% substitutions per site. Strains in bold letters are obtained in this study.

In the 16S rDNA phylogeny (Figure 1), the 25 representative strains were divided into two clades: 15 representative strains belonging to IGS types A and B were clustered with *S. medicae* WSM A321^T^ (99.9% similarity); while the other 10 representative strains belonging to IGS types C, D and E presented close relationships to *S. meliloti* USDA1002^T^ and *S. kummerowiae* CCBAU71714^T^ (>99.6% similarity; Figure 1). Similar relationships were obtained in the phylogenetic tree based on MLSA of the 5 concatenated housekeeping genes (Figure 2). Based on these results they were identified as *S. medicae* and *S. meliloti*. Even though plants of both *Medicago* species are compatible for these rhizobial stains, specific rhizobium-legume association were observed. *S. medicae* was predominant in *M. polymorpha* nodules (85.7%, 60/70), while *S. meliloti* was dominant in *M. lupulina* nodules (80.9%, 17/21).

### Phylogenies of symbiotic genes

**Figure 3:**
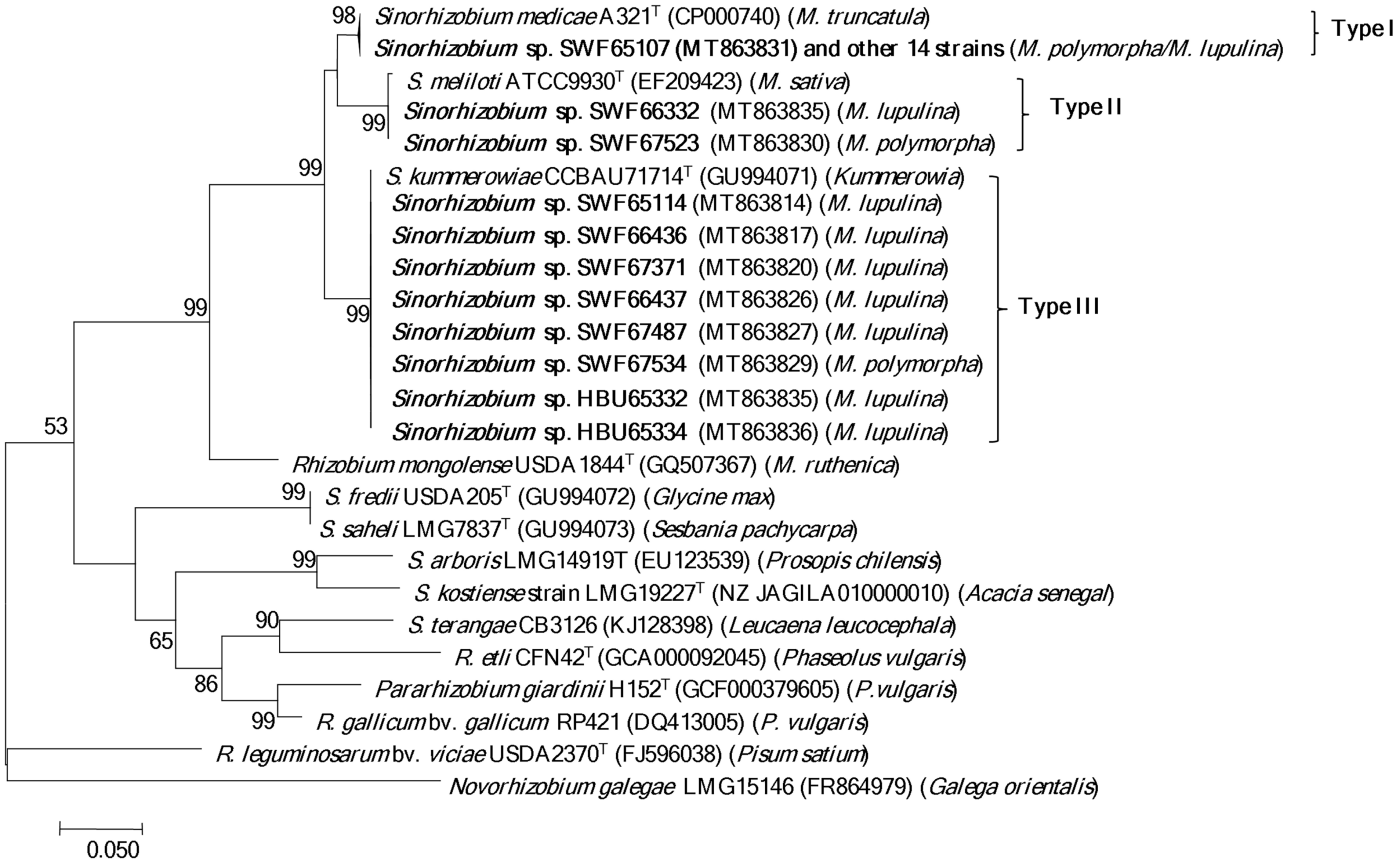
Phylogenetic tree of *nodC* genes (550 bp) showing the relationships among the rhizobia isolated from *Medicago* species in Yunnan. The tree was constructed with the Maximum likelihood method. Bootstrap values (>50% are indicated) in the nodes and the bootstrap analysis was performed with 1,000 replicates. Bar presents 5% substitutions per site. Strains in bold letters are obtained in this study.

In the phylogeny of *nodC* genes (Figure 3), the 15 *S. medicae* representative strains and the type strain *S. medicae* A321^T^ formed a group (type I) with almost identical sequences; while the 10 *S. meliloti* representatives were divided into 2 groups: SWF67523 and SWF66332 formed a group (type II) together with the type strain *S. meliloti* ATCC9930^T^ (=USDA1002^T^); and the other 8 representatives formed a monoclade (type III) together with *S. kummerowiae* CCBAU 71714^T^ at 100% identity. In line with the *nodC* phylogeny, the *S. meliloti* and *S. medicae* strains were also separated in the *nifH* phylogeny according to their species. Notably, all the *S. meliloti* strains tested in this study presented identical *nifH* sequences with *S. kummerowiae* CCBAU 71714^T^ and *S. meliloti* 1002^T^ (Supplementary Figure S7).

### Biogeographical patterns and genetic differentiation of *Medicago* rhizobia

**Figure 4:**
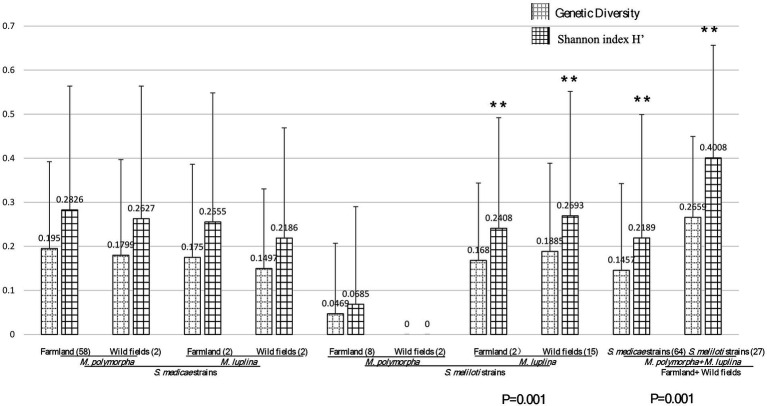
Diversity analysis between habitats gene diversity and Shannon index (H′) of rhizobial species from *M. polymorpha* and *M. lupulina* base on BOXAIR profiles. The marker** means the significant, the data in figure means the genetic diversity and Shannon index calculation result. *p* values of ANOVA analysis between habitats (farmland vs. wild field) are given below the bar plot.

The aforementioned phylogenic analysis indicated genetic incongruence in *S. meliloti* strains but not in *S. medicae* representatives. To know whether host specificity would affect genetic diversity of rhizobia in the studied area, we firstly compared the genetic diversity between the populations of both *Sinorhizobium* species. The *S. medicae* populations from *M. polymorpha* in both farmland and wild fields showed greater genetic diversity than that from *M. lupulina* nodules (H′ = 0.2826: 0.2555 in farmland; H′ = 0.2627: 0.2186 in wild fields) according to the Shannon index derived from BOXAIR-PCR patterns. Meanwhile, *S. meliloti* populations from *M. lupulina* showed significantly greater genetic diversity than that from *M. polymorpha* nodules (H′ = 0.0469: 0.168; 0: 0.2408; *p* = 0.001; Figure 4). These data suggested that rhizobial strains from their native host tend to be more genetically diverse. However, *S. medicae* populations from either of the hosts showed no significant difference in Shannon index and genetic diversity between the two ecosystems (*p* > 0.05), and neither *S. meliloti* populations did. Whereas, *S. meliloti* populations (from both legume species and ecosystems) showed significantly higher genetic diversity (0.1457: 0.2659) and Shannon index (0.2189: 0.4008) than the *S. medicae* counterparts (*p* = 0.001), indicating that the populations of these two rhizobial species have significant different beta-diversity. Moreover, a high level of genetic differentiation (Fst = 0.92054) was observed between *S. meliloti* and *S. medicae*, on the basis of the sequences of 5 housekeeping genes from the 25 representative strains (Table 3).

**Table 3:** Genetic diversity and differentiation analysis of concatenated keeping-house gene (16S rRNA-4genes) sequences of rhizobial species from two *Midicago* species.

Species	Habitat	Host	Nucleotide diversity estimates			Genetics differentiation and gene flow estimates			
			n	h	π	Kst*	P-value	Fst	Nm
*S. medicae*	Farmland	*M. polymorpha*	10	10	0.00413	0.01644	0.2150	−0.0109	−2.53
	Wild field	*M. polymorpha*	2	2	0.01366				
*S. meliloti*	Farmland	*M. lupulina*	3	3	0.00872	0.02607	0.9390	0.0255	1.87
	Wild field	*M. lupulina*	5	5	0.01076				
*S. medicae*	Farmland	*M. polymorpha*/*M. lupulina*	11	11	0.00416	0.01245	0.1310	0.0525	4.5
	Wild field	*M. polymorpha*/*M. lupulina*	4	4	0.01179				
*S. meliloti*	Farmland	*M. polymorpha*/*M. lupulina*	4	4	0.00848	0.00939	0.6630	0.0501	4.74
	Wild field	*M. polymorpha*/*M. lupulina*	6	6	0.01042				
*S. medicae*	Farmland/Wild field	*M. polymorpha*/*M. lupulina*	15	15	0.00441	0.31532	<0.0001***	0.9205	0.03
*S. meliloti*	Farmland/Wild field	*M. polymorpha*/*M. lupulina*	10	10	0.00820				
*S. medicae*	Farmland	*M. polymorpha*	10	10	0.00431	0.29381	<0.0001***	0.9081	0.02
*S. meliloti*	Wild field	*M. lupulina*	5	5	0.01076				

n, Number of sequences; h, Number of haplotypes; π, Nucleotide diversity per site; Nm, the minimum number of migration events. Fst, F-statistics of the genetic differences among population. For Fst, the range 0.0 to 0.05 may be considered as indicating little genetic differentiation; 0.05 to 0.15, moderate genetic differentiation; 0.15 to 0.25, great genetic differentiation; above 0.25 indicate very great genetic differentiation. For Kst*, if the observed value of the statistic had *P*-value < 0.05, the null hypothesis of no genetic differentiation was rejected. Probability obtained by the Monte Carlo permutation test with 1,000 replicates. The symbols * and *** means different significant values.

**Figure 5:**
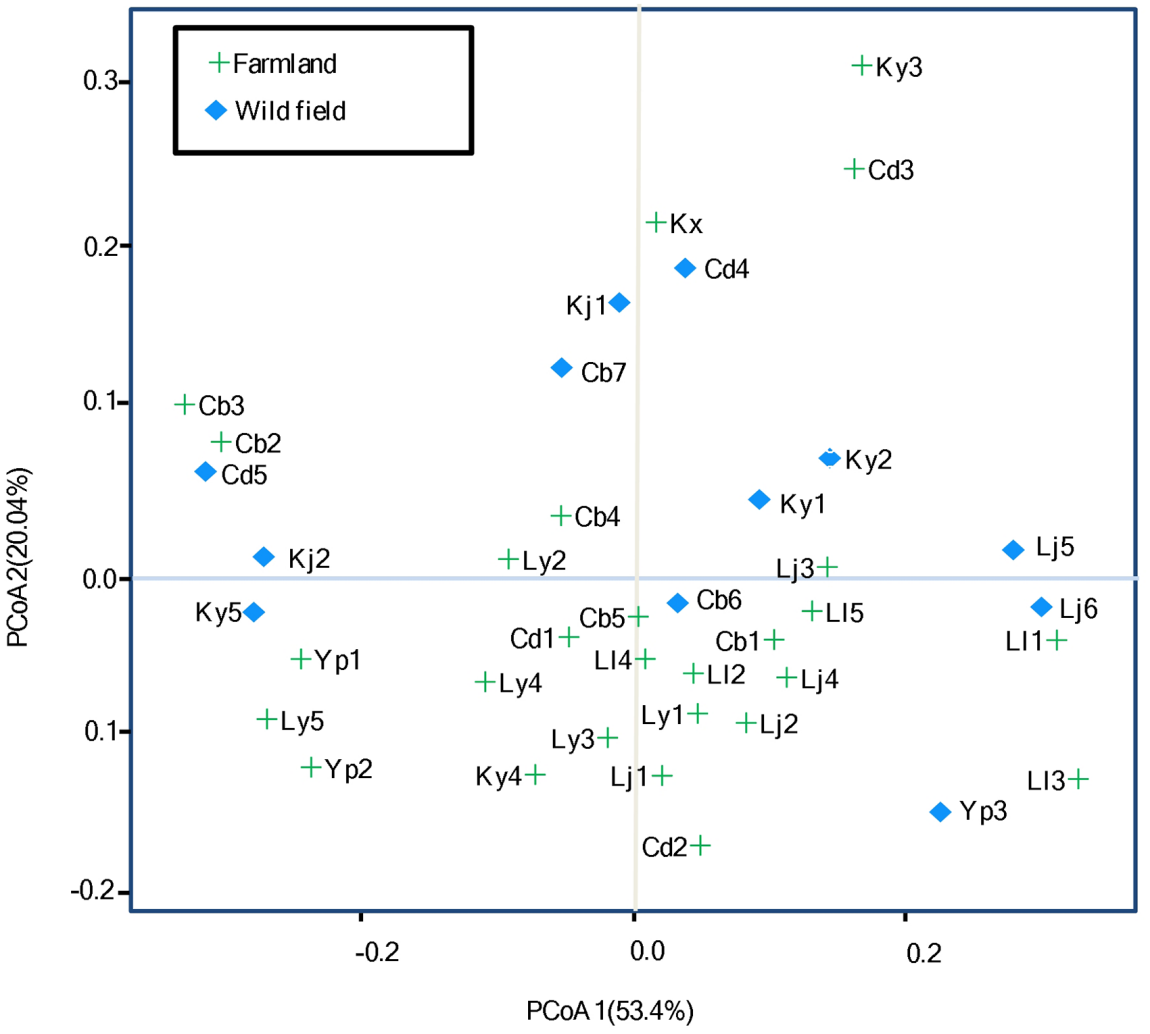
Principal coordinates analysis (PCoA) based on Bray-Curtis distance. The percentage of contribution to the variance (inertia) of the rhizobial diversity is given in parenthesis. See Supplementary Table S2 for detail for site codes in farmland and wild fields.

The effects of habitats on rhizobial diversity were evaluated by the analysis of PCoA based on Bray-Curtis distance (Figure 5; Supplementary Table S3). Although, the amount of multiple nutrient factors extensively varied among the sites in wild fields, while it tended to be less divergent in the farmland sites, as the observation that the component 1 represented 53.4% of relative eigenvalues, and component 2 represented 20.04% of relative eigenvalues (after Cailliez correction; Figure 5), these two ecosystems were separated along the second principal coordinate, with 8/12 of the wild field sites displayed on the upper side of PcoA 2, and 19/27 of the farmland sites displayed on the lower side. Furthermore, multivariate analysis of variance (MANOVA) revealed that the *S. medicae* distribution significantly differed between the two ecosystems (*p* = 0.047). These observations demonstrated that rhizobial species variation is relative to the land utilization; while no overt difference was observed for *S. meliloti* populations between farmland and wild fields. Permutational multivariate analysis of variance (PERMANOVA) corroborated that soils in farmland and in wild fields accounted for 20.66% (*p* = 0.001) of variation in the observed beta-diversity of rhizobia (Bray-Curtis distance metric).

On one hand, all the 10 *Sinorhizobium* strains originated from wild fields had a higher level of nucleotide diversity (π) than those from farmland (Table 3), suggesting that the characters of ecosystems might contribute to rhizobial genetic diversity. The greater variation in the content of several nutrients in wild fields might explain the higher genetic diversity of rhizobia. Consistently, moderate level of genetic differentiation was observed within the *S. medicae* populations from both legumes (Fst = 0.05251) or the *S. meliloti* populations from these two legume species (Fst = 0.0501) between the two ecosystems. No genetic differentiation was detected among the strains from the same legumes in the two ecosystems. Furthermore, gene flow was detected for *S. meliloti* (Nm = 4.74) between the two ecosystems, and the *S. medicae* (Nm = 4.50) strains did; while almost no gene flow was observed between strains of the two rhizobial species in any growth habitats (Table 3).

### Deterministic factors for diversity of rhizobia from two *Medicago* plants

**Figure 6:**
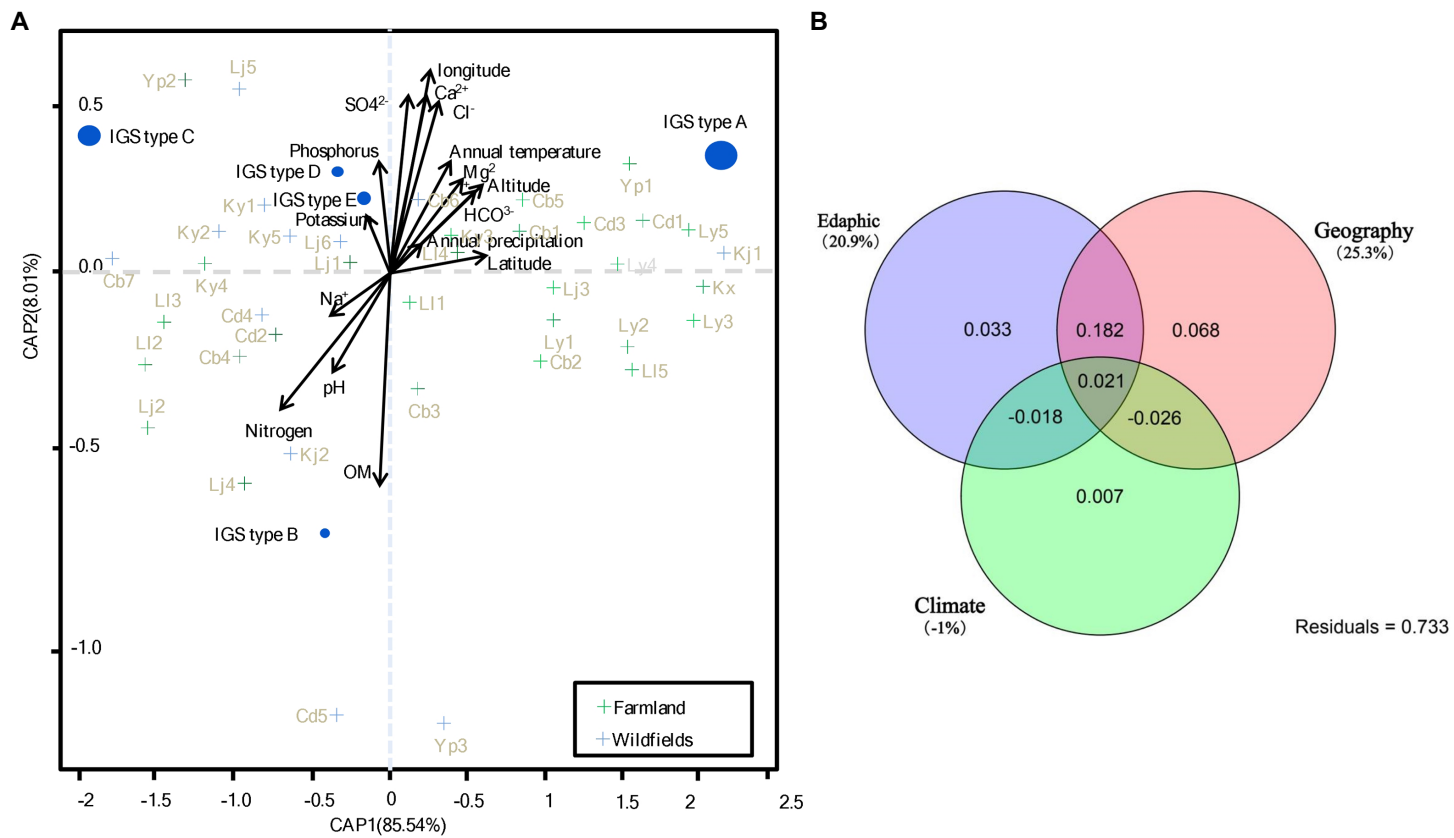
CAP (constrained analysis of principle coordinates) and variation partition analysis. **(A)** CAP analysis based on Bray-Curtis distance of Hellinger transformed rhizobial species data. The size of species points is scaled to the square transformed mean abundance. **(B)** Variation partition analysis based on CAP analysis in **(A)**. The percentage of contribution to the variance (inertia) of the species are given in parenthesis of **(A)**. *p* values of Monte Carlo permutation test are given for all statistical an alyses (9,999 permutations). See Supplementary Table S2 for detail for site codes in farmland and wild fields.

To identify the soil factors that determine the genetic diversity of rhizobial populations, analysis of variance (ANOVA) based on a set of environmental factors was performed. Compared to the wild fields, the alfalfa farmland had a significantly higher contents of multiple nutrients and minerals, including organic matter (OM), sodium (Na^+^), magnesium (Mg^2+^) and bicarbonate (HCO_3_^−^; Supplementary Tables S4, S5). As shown in the CAP analysis based on Bray-Curtis distance of PA (rhizobial abundance) transformed species data (Figure 6A), soil contents of OM, nitrogen, Ca^2+^, Cl^−^ and SO_4_^2−^, as well as the longitude were the most important factors (according to the arrow length) to affect the distribution of rhizobial populations (IGS types) in this study. The contents of phosphorus, Mg^2+^ and HCO_3_^−^, as well as annual temperature, altitude, and pH contributed moderately to the biogeography of rhizobia; while the contents of Na^+^, K^+^ and annual precipitation presented less effects on rhizobial distribution in this study.

Rhizobial species correlated with different sets of explanatory variables were further identified in Figure 6. IGS type A of *S. medicae* was more likely found in the farmland sites with higher annual temperature, HCO^3−^ and Mg^2+^ contents, latitude, and altitude, but lower values of Na^+^, nitrogen and pH; while IGS type B of the same species was in contrast. IGS type C (*S. meliloti*) was more likely found in the wild fields with high soil K^+^ and phosphorus contents. The IGS types D and E more likely appeared in the wild field sites similar with IGS type C, but they were not correlated to Na^+^ content. In summary, IGS types A and B in *S. medicae* have opposed nutrition requirements, while the factors for distribution of *S. meliloti* IGS types were intermediate between the types A and B, but more related to K^+^ and phosphorus contents. IGS types A (major group of *S. medicae*) preferred acid soils with low nitrogen, Na^+^, and high HCO^3−^, growing in high latitude locations; while IGS type C (major group of *S*. *meliloti*) preferred alkaline soil with high contents of Na^+^ and low HCO^3−^, also tend to perch in low latitude sites (Figure 6A). Variation partition analysis revealed that 20.9 and 25.3% could be significantly explained by edaphic and geographic variables, respectively; while the climate factors showed no contribution to rhizobial species distribution (Figure 6B).

## Discussion

In the present study, we systematically investigated the diversity of rhizobia associated with *M. polymorpha* in comparison with those associated with *M*. *lupulina* grown in the same region (Yunnan Province). Based upon the results of BOXAIR-PCR patterns, IGS-RFLP and MLSA, the rhizobia from both *Medicago* species were identified as diverse populations belonging to *S. medicae* and *S. meliloti*. The species affiliation in this study was mainly based on the MLSA analysis (Figure 2), in which 95% similarity (de Lajudie et al., 1994) or 97% similarity (Tong et al., 2018) as species threshold was taken. Similar to previous reports, the phylogeny of 16S rRNA gene (Figure 1) was unable to distinguish the closely related species (de Lajudie et al., 1994; Tong et al., 2018). The high similarity in MLSA between the type strains of *S. meliloti* and *S. kummerowiae* examined in the present study was similar to the previous reports (Yan et al., 2016; Chen et al., 2017), indicating that the independent species state of *S. kummerowiae* needs further reversion.

In accordance with previous reports (Brunel et al., 1996; Garau et al., 2005; Silva et al., 2007), dominance of *S. medicae* associated with *M. polymorha* (86.7% nodule occupation), and of *S. meliloti* with *M. lupulina* (80.95% of nodule occupation) was observed. The preference of *S. medicae* by *M. polymorpha* was rather apparent in farmland (58/8, *S. medicae/S. meliloti*) than wild fields (2/2, *S. medicae/S. meliloti*). Given that all the *S. medicae* strains harbor the same symbiotic genes (*nodC* and *nifH*; Figure 3; Supplementary Figure S7), the difference in relative abundances of *S. medicae* in *M. polymorpha* might be mainly related to the soil characters in the two ecosystems. Purple soils are widely distributed in rice field in present study, in which the inorganic N can be easily lost in the rainy climate (Zhao et al., 2018). Thus low nitrogen status allows medics to attract more efficient rhizobial symbiosis to fix nitrogen.

Furthermore, the results of isolation and nodulation tests evidenced that both the *Medicago* species could be nodulated by both the *S. meliloti* and *S. meliloti* strains, indicating *M. polymorpha* and *M. lupulina* belong to the same cross-nodulation group. However, similar to previous findings (Garau et al., 2005; Villegas et al., 2006; Liu et al., 2011; Alías-Villegas et al., 2015), *S. medicae* strains were more efficient microsymbionts for *M. polymorpha* as contrast to *S. meliloti*. Additionally, distinct *nodC* and *nifH* types were detected between the *S. medicae* strains (*nodC* type I and *nifH* type I) and the *S. meliloti* strains (*nodC* types II and III, *nifH* type II) (Figure 3; Supplementary Figure S7), demonstrating that the chromosome and symbiotic genes in each of the two *Sinorhizobium* species have co-evolved. A similar case is the soybean nodulating rhizobia in northern China, for which four rhizobial species *Sinorhizobium fredii*, *Bradyrhizobium japonicum*, *Bradyrhizobium yuanmingense* and *Bradyrhizobium elkani* harbor different symbiosis genes (Li et al., 2011; Zhang et al., 2011). The co-evolution might be related to a geographic and/or host selection, together - forcing the two closely related species evolved divergently to adapt to their local conditions. Of note, the *nodC* type III is relatively distinct from the other two, indicative of a possible native lineage in the studied area or co-evolved lineage together with *M. lupulina* in Yunnan. The inclusion of *S. kummerowiae* type strain in *nodC* type III might imply that *Kummerowia* species and *M. lupulina* could share their microsymbionts. The grouping of *nodC* types II and III strain in the same *nifH* lineage supported the previous observation that *nif* and *nod* genes might have different evolutionary route in some rhizobia (Okubo et al., 2016). This phenomenon was also discovered in beta-rhizobia isolated from *Mimosa* in our previous work (Liu et al., 2020). Moreover, rhizobia containing different *nodC* genes might synthesize Nod factors with different structures, which may affect rhizobial symbiotic performance in nodulation and infection. In present study, two *S. meliloti* strains (SWF66332 and SWF67523) harboring the type II *nodC*, showed weaker nodulation capacity, indicating that this *nodC* type might not be optimal for the nodulation of *M. polymorpha*. Considering the fact that only three of the 91 strains harbored the type II *nodC,* it might be evidence that the incompatible symbiosis genes have suffered the host sanction, therefore only maintained a low relative abundance in the rhizobial population.

The isolation of both *S. medicae* and *S. meliloti* from the two studied *Medicago* species, as well as their cross nodulation in our study demonstrated that the specificity between these two *Medicago* species and their microsymbionts appeared not so stringent, although *M. polymorpha* was recognized as the sole host of *S. medicae* in some areas (Brunel et al., 1996; Rome et al., 1996; Biondi et al., 2003). Therefore, it could be estimated that the associations between the hosts *M. polymorpha*/.

*M. lupulina* and their microsymbionts *S. medicae*/*S. meliloti* were not only determined by the preference (or specificity) between the hosts and the rhizobia, but were also regulated by the soil abiotic and biotic conditions, like the cases of *Mesorhizobium* associated with chickpea (*Cicer arietinum* L.; Zhang et al., 2014) and the soybean nodulating rhizobia (Li et al., 2011; Zhang et al., 2011). However, the sub-optimal efficiency in nitrogen fixation and lower abundance of *S. meliloti* strains in *M. polymorpha* nodules in our work (Supplementary Table S2) and in other studies (Garau et al., 2005; Villegas et al., 2006; Alías-Villegas et al., 2015) evidenced that *S. meliloti* is not an optimal microsymbiont for *M. polymorpha.*

In this study, the predominance of *S. medicae* in farmland for *M. polymorpha* and *S. meliloti* in wild fields for *M. lupulina* was recorded and the biogeographic pattern for these two rhizobial species was evidenced. *S. medicae* displayed strong negative correlation to soil pH and nitrogen at present study, while *S. meliloti* positively correlated with pH. These correlations were consistent with the work of Garau et al. (2005) which reported *S. medicae* as the microsymbiont associated with most medics grown in moderately acid soils, such as *M. polymorpha*, *M. arabica* and *M. murex*; whereas *S. meliloti* was predominantly isolated from *M. littoralis* and *M. tornata* naturally grown in soils with alkaline or neutral pH, and from *M. sativa* (Ramírez-Bahena et al., 2015). In our present study, both *S. medicae* and *S. meliloti* strains were isolated from soils ranging from slight acid (pH5.5) to slight alkaline (pH7.8) with most from slight alkaline soils. Taken together, *S. medicae* could adapt to slightly alkaline soil in Yunnan, but it still preferred soils with lower pH when compared to the *S. meliloti* strains isolated from the same region (Figure 6).

In accordance with another report (Bailly et al., 2007), our results demonstrated that the soil type (or ecological environment) have effect on rhizobial diversity. The PCoA results (Figure 5) evidenced that *S. meliloti* distributed geographically more scattered than *S. medicae*, and it might be related to the fact that *M. polymorpha* was mostly distributed in farmlands with similar soil traits, while *M. lupulina* distributed in more divergent wild fields (Supplementary Table S3; Supplementary Figure S1). In this study, only *S. meliloti* strains were isolated from *M. polymorpha* in Yinjiang (both in farm land and in wild fields), while the remaining *S. meliloti* strains were recovered from *M. lupulina* in other sites (Table 1). In addition, both *S. meliloti* and *S. medicae* were isolated from *M. lupulina* in Yiliang (both in farm land and in wild fields). These data further evidenced that both the hosts and geographical factors deeply influenced the distribution of rhizobia (Chen et al., 2004; Béna et al., 2005; Wang et al., 2019).

Based on the BOXAIR fingerprinting profiles of all the 91 isolates, *S. meliloti* populations showed significantly high level of genetic diversity than the *S. medicae* counterparts associated with both hosts and in both ecosystems in Yunnan. Analysis on the nucleotide diversity of 25 representatives also supported this observation. However, this situation needs further study since the difference may be due to the unbalance in strain numbers from different plant species and from different ecosystems in the analysis. Furthermore, the rhizobial strains tested in this study (for both *Sinorhizobium* species strains) from wild fields have higher nucleotide diversity than those from farmland. This suggested that *Medicago* symbionts might undergo more gene exchange events with other bacteria in the wild fields compared to that in the farmland. Indeed, a greater value of genetic differentiation was observed at the interspecies level compared to the intraspecies level, implying that these two species have divergence evolution, and genome analysis also stated it (Sugawara et al., 2013). *S. meliloti* was more frequently recovered from wild fields; while *S. medicae* strains were more readily isolated from *M. polymorha* in farmland in this study. These two *Sinorhizobium* species retained their own genetic characteristics. We found high levels of gene flow (Nm) within *S. medicae* or *S. meliloti* populations in the two ecosystems. Various subpopulations of the same *Sinorhizobium* species could intermingle their genes through the horizontal gene transfer processes such as conjugation and transformation, which conferred sufficient genetic exchanges among populations (Andrews et al., 2018).

To conclude, both *S. medicae* and *S. meliloti* strains could naturally nodulate with both the tested Medics in purple soil of Southern China, with *S. medicae* dominant in *M. polymorpha* nodules and *S. meliloti* dominant in *M. lupulina* nodules. The diversity and distribution of rhizobia associated with the *Medicago* species were regulated by both the host specificity and soil factors in Yunnan. Three *nodC* types were defined among the strains, including two corresponding to that of *S. meliloti* and *S. medicae* type strains, and a unique type only found in *S. meliloti* strains in Yunnan, which might be a result of adaptation to the dual selection by host plants and soil traits.

## Data availability statement

The datasets presented in this study can be found in online repositories. The names of the repository/repositories and accession number(s) can be found in the article/Supplementary material.

## Author contributions

MT: original writing and collection and isolation rhizobia. HW: original draft preparation and software and data curation. XQ: original writing and software. TH: software and data curation. MY: phylogenetic analysis. BZ: collection and isolation strains. EW: supervision and writing—reviewing and editing. BW: gene amplifying and data curation. FW: conservation of strains. ZL: conceptualization, writing, and visualization. XL: conceptualization, methodology, and writing. All authors contributed to the article and approved the submitted version.

## Funding

This research was financially supported by China National Modern Agricultural Industrial Technology System (CARS-34), National Natural Science Foundation of China (grant nos. 31370051 and 31360003), China Agricultural Research System-Green Manure (CARS-22), Key Discipline Project for Biotechnology of Hebei Province, and First-Class Discipline Construction Funding of Hebei Province.

## Conflict of interest

The authors declare that the research was conducted in the absence of any commercial or financial relationships that could be construed as a potential conflict of interest.

## Publisher’s note

All claims expressed in this article are solely those of the authors and do not necessarily represent those of their affiliated organizations, or those of the publisher, the editors and the reviewers. Any product that may be evaluated in this article, or claim that may be made by its manufacturer, is not guaranteed or endorsed by the publisher.
