# Supplementary figures and images for: Diversification of Sinorhizobium populations associated with Medicago polymorpha and Medicago lupulina in purple soil of China
Source: Front Microbiol. 2023 Jan 4;13:1055694. doi: 10.3389/fmicb.2022.1055694 (PMC9846747; doi:10.3389/fmicb.2022.1055694)

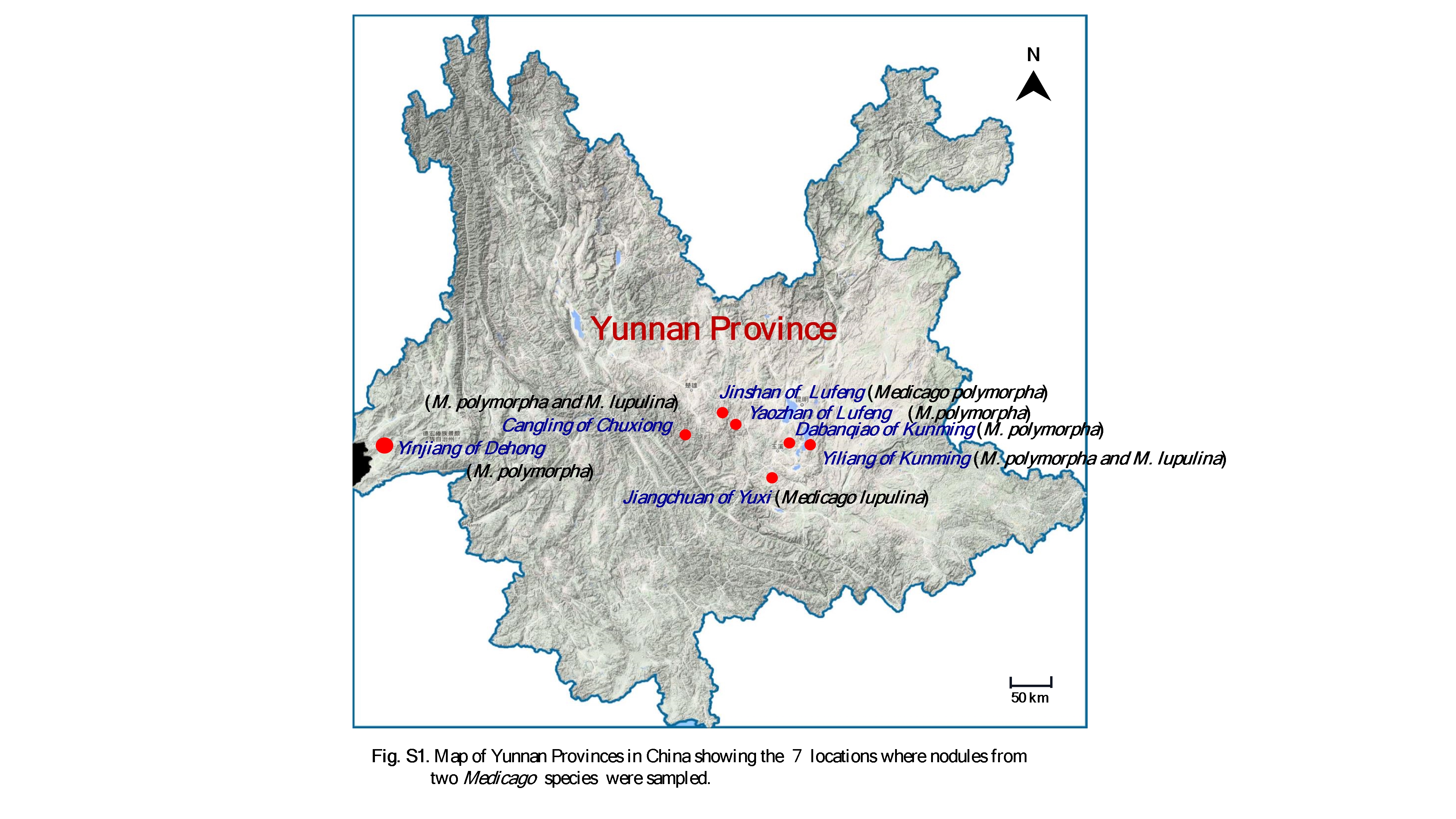

Supplement: Supplementary file 1 [file Image_1.JPEG]

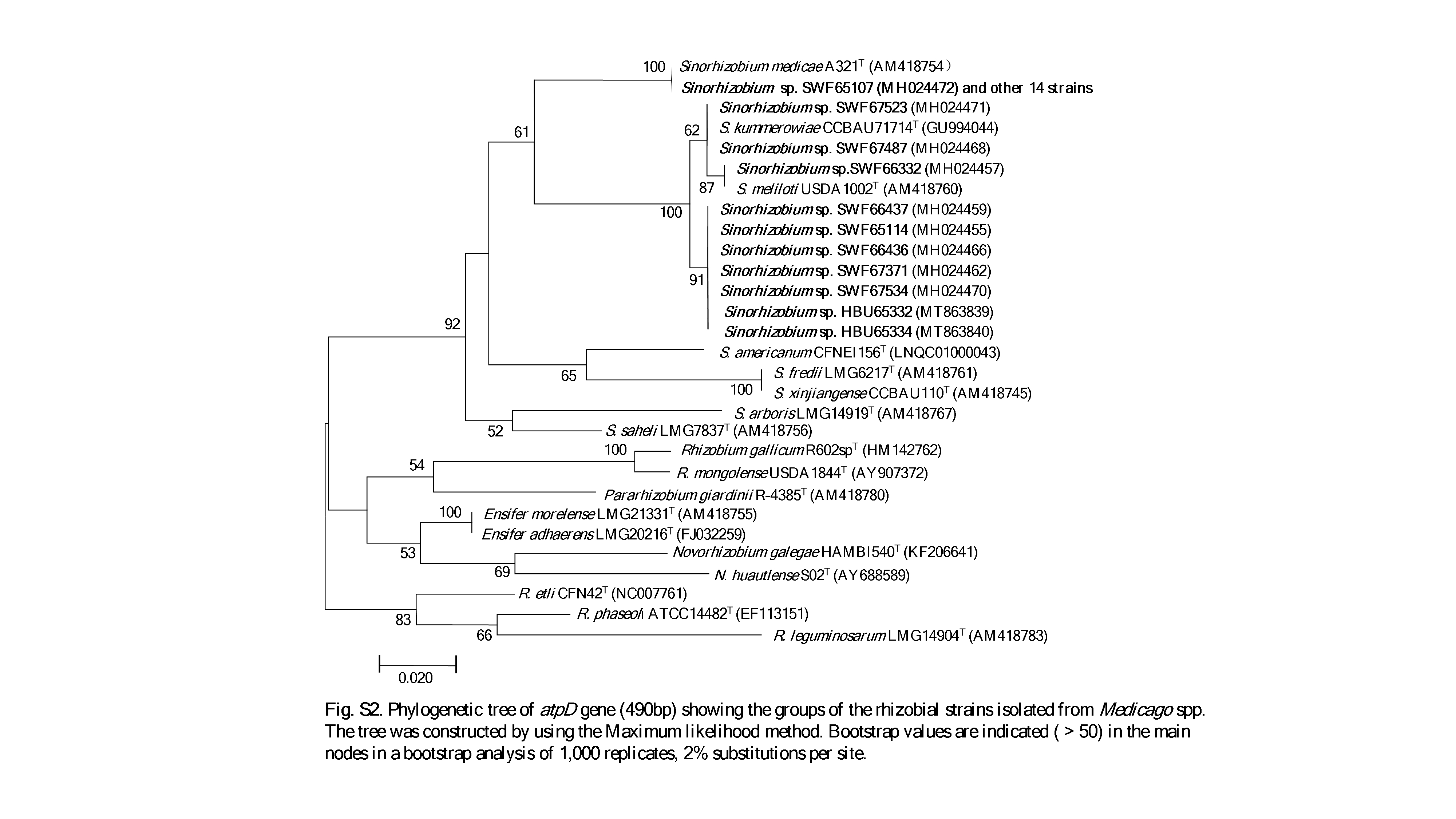

Supplement: Supplementary file 2 [file Image_2.JPEG]

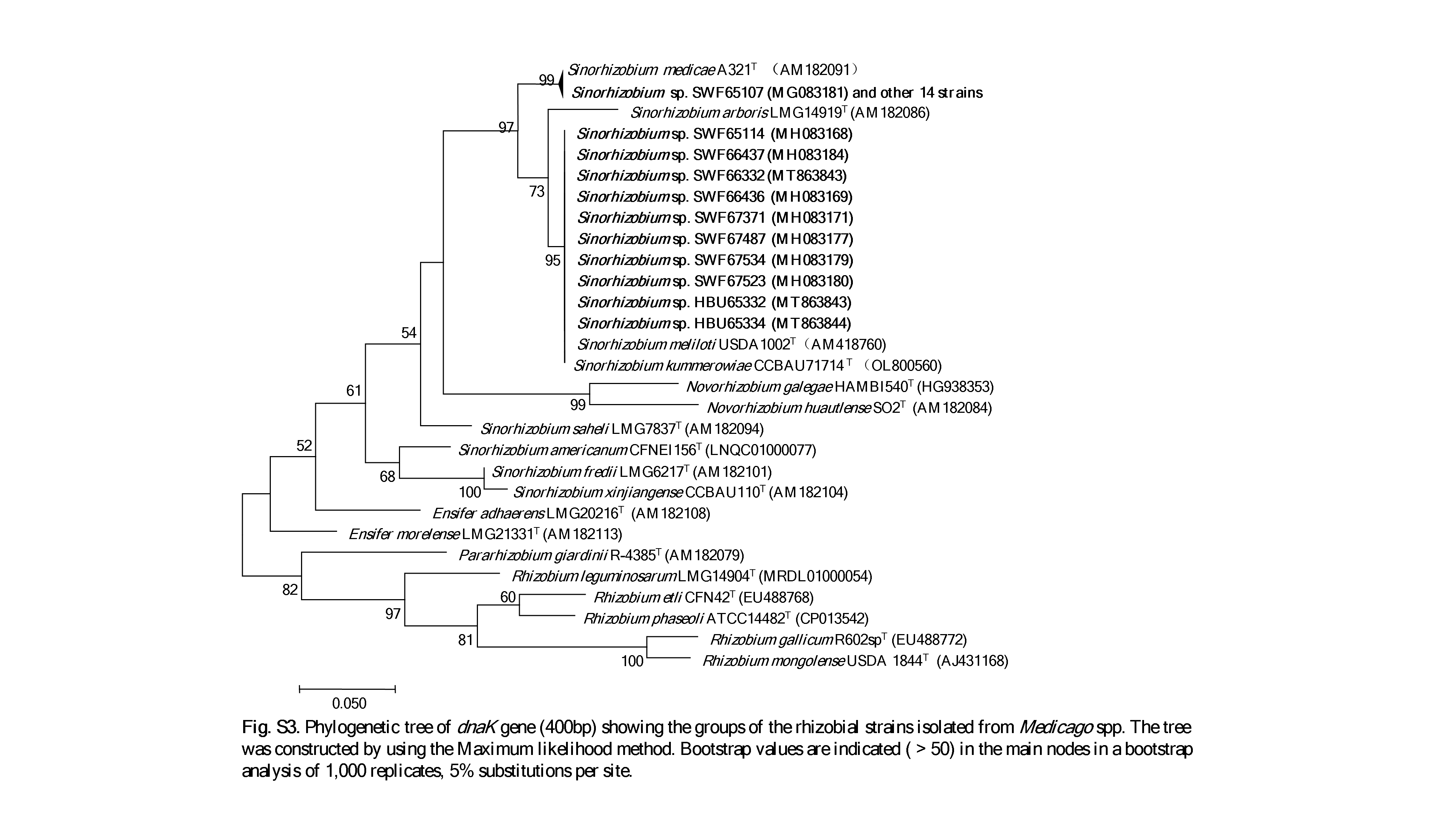

Supplement: Supplementary file 3 [file Image_3.JPEG]

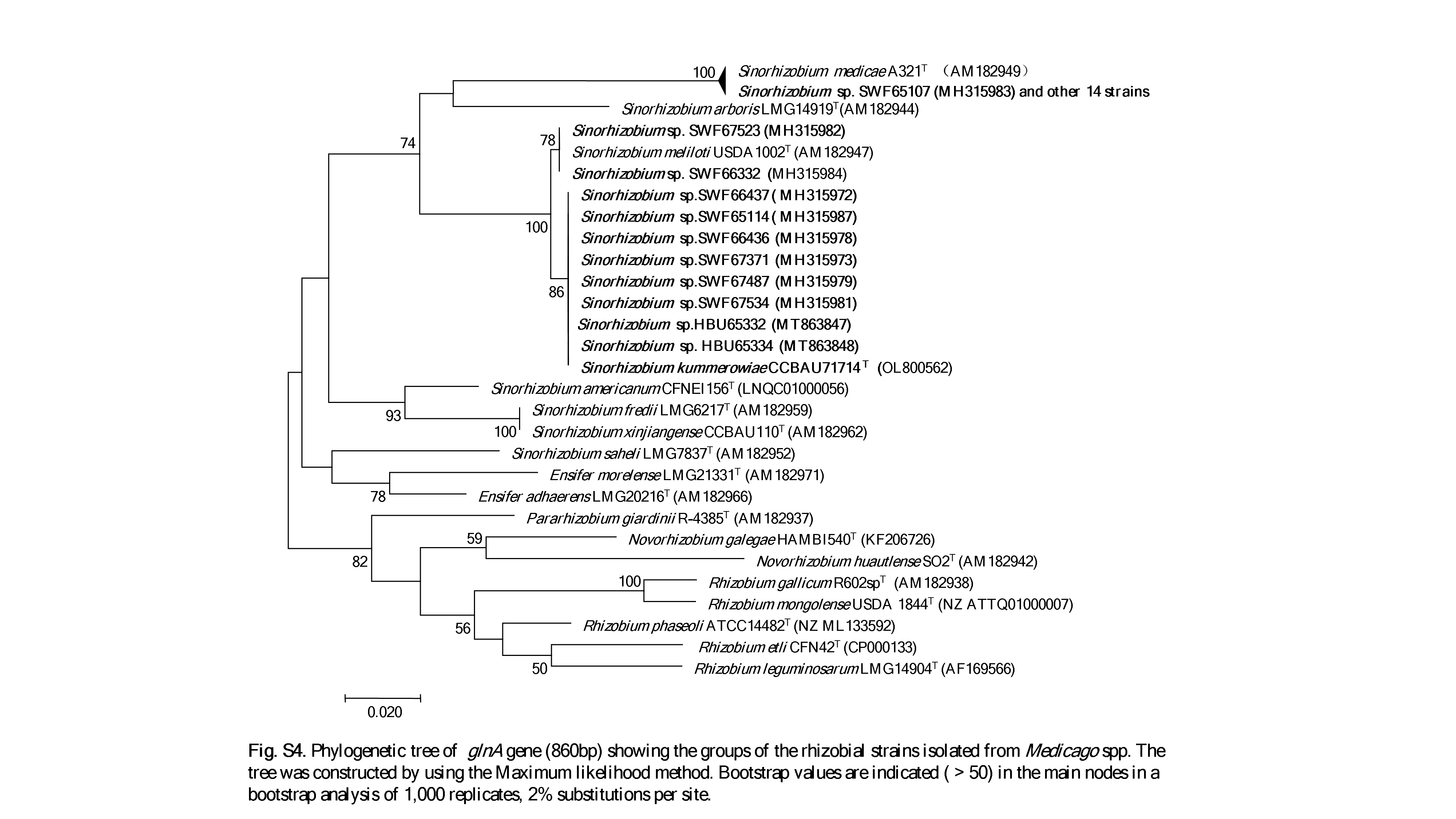

Supplement: Supplementary file 4 [file Image_4.JPEG]

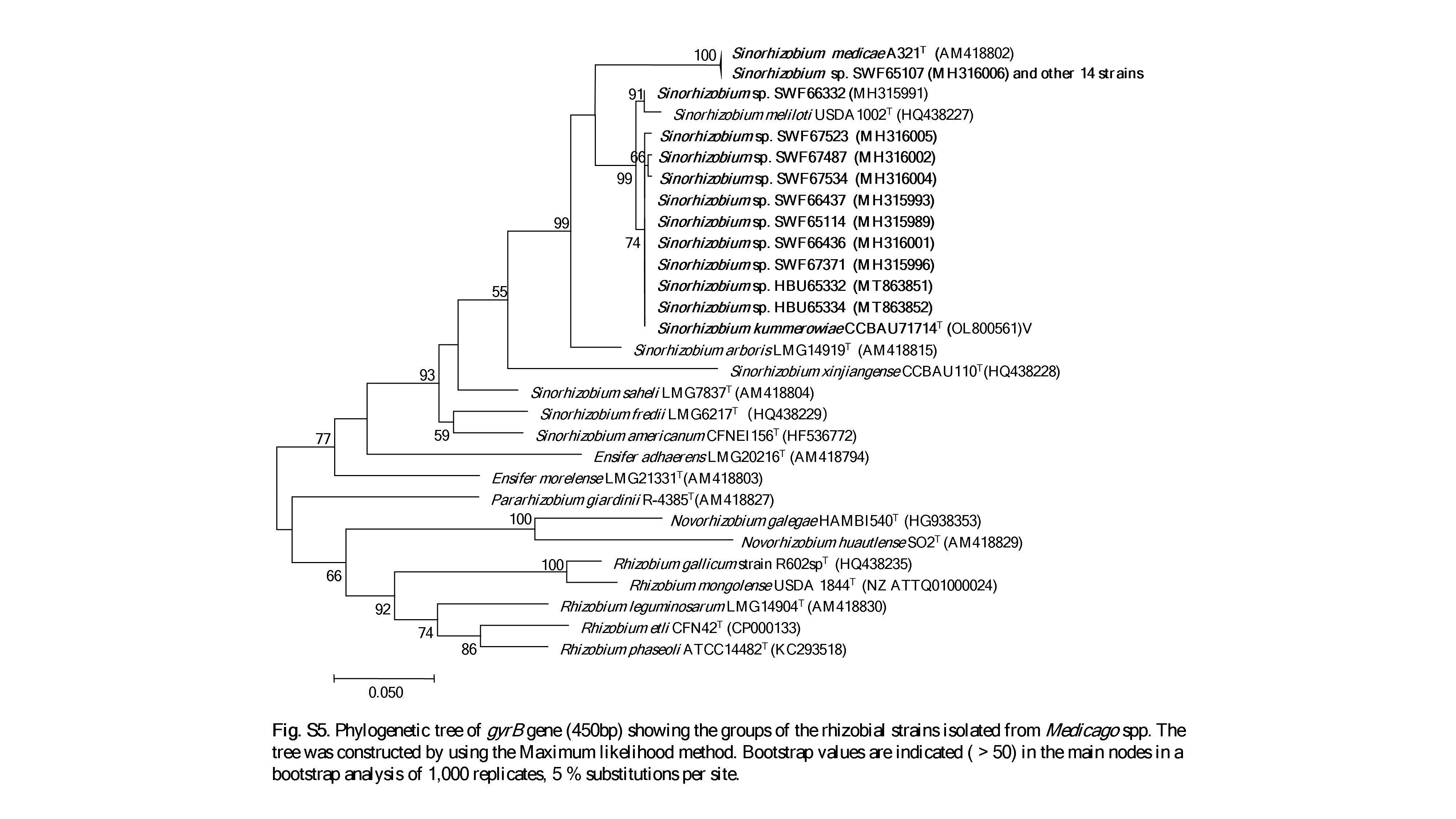

Supplement: Supplementary file 5 [file Image_5.JPEG]

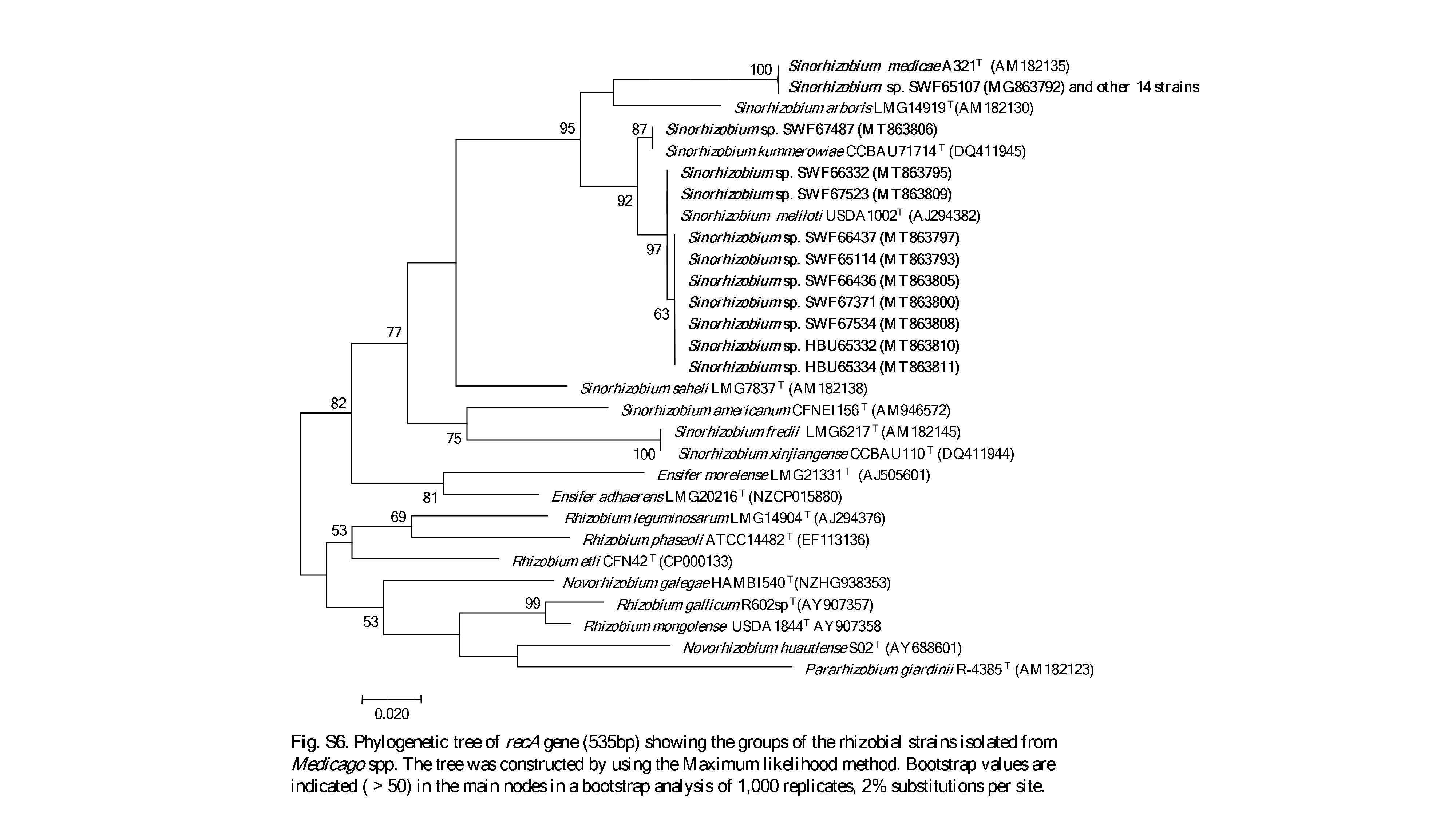

Supplement: Supplementary file 6 [file Image_6.JPEG]

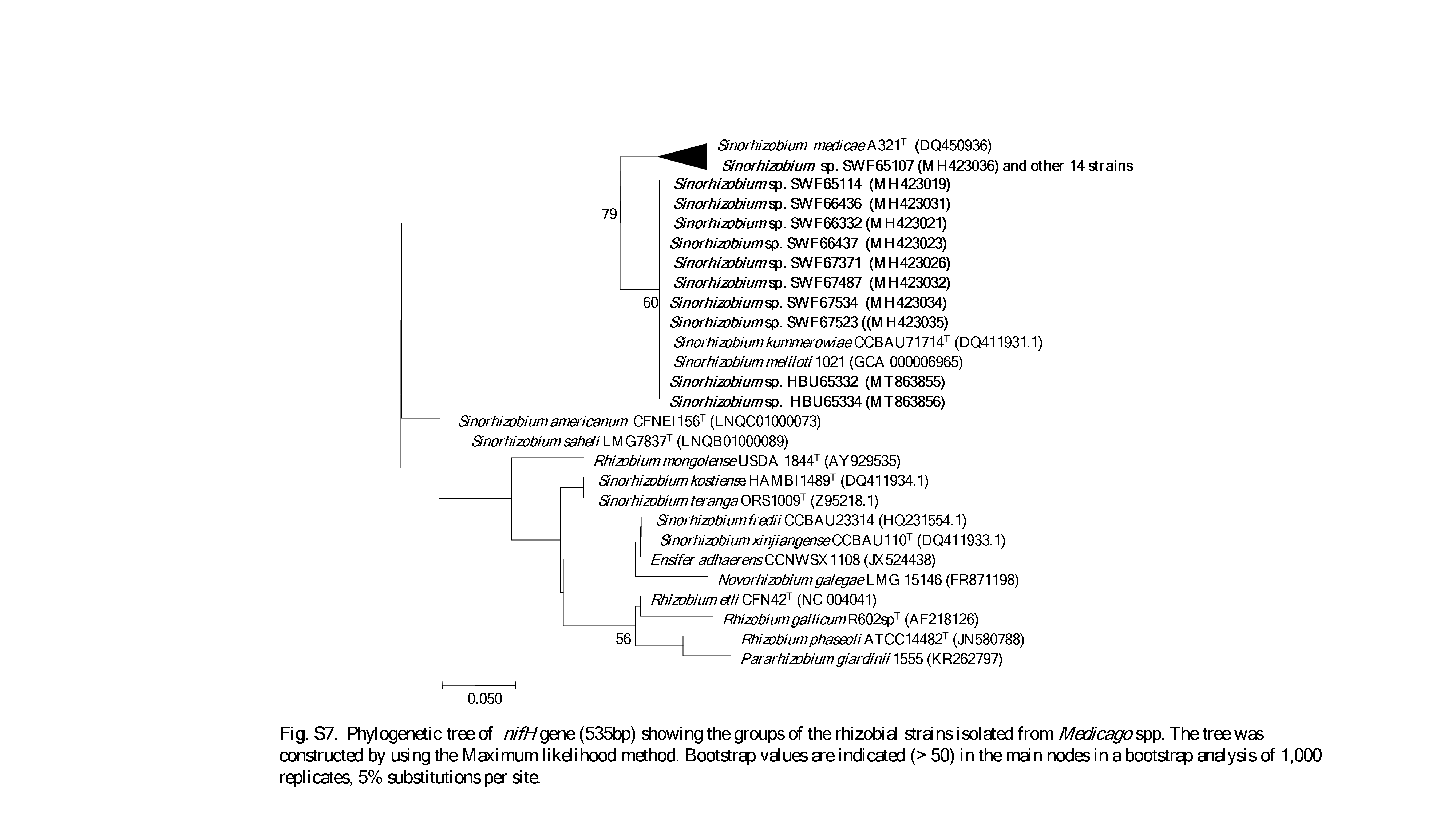

Supplement: Supplementary file 7 [file Image_7.JPEG]
